# Nylon-flocked swab collection method better predicts high-grade AIN than does dacron swab method

**DOI:** 10.1186/1750-9378-7-S1-P49

**Published:** 2012-04-19

**Authors:** Dorothy Wiley, Robert Bolan, Alen Voskanian, Stephen Young, David Elashoff, Hilary Hsu, Emmanuel Masongsong, Provaboti Barman, Roger Detels

**Affiliations:** 1 School of Nursing, UCLA, Los Angeles, CA, USA; 2 L.A. Gay & Lesbian Center, Los Angeles, CA, USA; 3 School of Medicine, UCLA, Los Angeles, CA, USA; 4 Tricore Diagnostic Laboratories, University of New Mexico, Albuquerque, NM, USA; 5 Institute of Geophysics and Planetary Physics, UCLA, Los Angeles, CA, USA; 6 School of Public Health, UCLA, Los Angeles, CA, USA

## Background

Invasive anal cancer (IAC) is a health crisis for gay, bisexual, transgender, and other men who have sex with men (MSM) who show a 20-40 fold higher risk for disease, especially if infected by HIV despite the introduction of HAART. *Human papillomaviruses* (HPV) that cause invasive cervical cancers (ICC) in women appear responsible for the majority of IACs. Although cervical cytology using Pap test has reduced ICC incidence by ~70%, anal Pap test only shows modest sensitivity and poor-to-modest specificity for detecting high-grade anal intraepithelial neoplasias (HG-AIN). Currently anal Pap testing using Dacron swab is recommended annually and biennially for HIV-infected and uninfected MSM, respectively. Swabs are inserted blindly through the anus, and ASCUS, low- and high-grade dysplasias (LG-, HG-SIL) are evaluated using high resolution anoscopy (HRA).

## Material and methods

Dacron-swab cytology specimens were collected first using standard procedures; subsequently, Nylon Flocked (NF)-swabs were collected through an anoscope inserted just beyond the verge. Swabs were approximated to the canal, rotated slowly while withdrawn, and placed into preservatives. HRA, with medical biopsy, where indicated, was performed by experienced clinicians. Pathologists evaluated cytology using the Bethesda Classification System, and histology using the International Classification of Diseases for Oncology. HPV genotypes were assessed from cytology specimens using Linear Array (Roche Diagnostic Laboratories, Pleasanton, CA).

## Results

Among 69 specimens obtained, 10 Dacron and 8 NF-specimens were inadequate for cytological evaluation: 14.5% and 11.6%. Sensitivity for HG-AIN and specificity were higher for cytology using NF- than Dacron swabs: 82% (66-98%) and 59% (44-74%), versus 55% (34-76%) and 49% (33-65%), respectively. Multivariate analyses showed NF-swab specimens more accurately predicted HG-AIN than Dacron swabs. Specimens showing either ASCUS /LG-SIL, or HG-SIL on NF-swab were 10 (1.9, 52.0) and 5.3 (0.4, 74) times more likely than unaffected specimens similarly collected to predict HG-AIN; whereas, Dacron-swab specimens using these cut-points showed no statistically greater risk for HG-AIN on histology, OR=0.4 (0.1, 2.3) and OR=4.7 (0.4, 61.5), respectively. These relationships persisted after controlling for age, HIV-infection, duration of infection, and multiple observations (n=7).

## Conclusions

Cytology specimens using Dacron swab blindly inserted through the anus less often predicted HG-AIN than did NF-swab specimen used in conjunction with an anoscope to guide placement.

